# The maximum standardized FDG uptake on PET-CT in patients with non-small cell lung cancer

**DOI:** 10.1186/2049-6958-8-69

**Published:** 2013-10-22

**Authors:** Mehmet Akif Özgül, Gamze Kirkil, Ekrem Cengiz Seyhan, Erdoğan Çetinkaya, Güler Özgül, Mahmut Yüksel

**Affiliations:** 1 Department of Chest Disease, Yedikule Chest Disease and Thoracic Surgery Education and Research Hospital, Istanbul, Turkey; 2 Department of Chest Disease, Firat University Hospital, Elazig, Turkey; 3 Department of Chest Diseases, Istanbul Medipol University Hospital, Istanbul, Turkey; 4 Medical Park Hospital, Istanbul, Turkey

**Keywords:** Non-small cell lung cancer, Positron emission tomography, Standardized uptake value

## Abstract

**Background:**

Non-small cell lung cancer (NSCLC) accounts for approximately 80% of new diagnoses of pulmonary carcinoma. This study investigated the correlation between 18 F-fluorodeoxyglucose uptake in computerized tomography integrated positron emission tomography and tumor size, lymph node metastasis, and distant metastasis in patients with NSCLC.

**Methods:**

The records of 151 NSCLC patients (139 male, 12 female; mean age 59.60 years) were evaluated retrospectively.

**Results:**

Forty-one cases were adenocarcinomas; 45 squamous cell carcinomas; and 65 unspecified NSCLC. When the cases were categorized according to tumor size (group 1, ≤ 3 cm; group 2, > 3 and ≤ 5 cm; group 3, > 5 cm), the maximum standardized uptake value (SUVmax) was significantly lower in groups 1 and 2 compared with group 3 (p = 0.006 for each). Considering all cases, tumor SUVmax was not correlated with age, gender, or histopathological type. Lymph node metastases were pathologically proven in 24 cases: 24% of these were adenocarcinomas, 6% squamous cell carcinomas, and 16% unspecified NSCLC. Neither lymph node involvement nor distant metastases were correlated with tumor SUVmax, although lymph node size was positively correlated with lymph node SUVmax (r = 0.775; p < 0.001).

**Conclusions:**

SUVmax was significantly associated with tumor size, but not with distant metastases or lymph node involvement. Therefore, SUVmax on positron emission tomography is not predictive of the presence of metastases.

## Background

Pulmonary carcinoma is the most commonly diagnosed cancer worldwide (1.61 million cases, 12.7% of total carcinomas) and is the most common cause of cancer death (1.38 million deaths, 18.2% of total cancer deaths) [1]. Non-small cell lung cancer (NSCLC) accounts for approximately 80% of new pulmonary carcinoma diagnoses and includes the histological subtypes adenocarcinoma, squamous cell carcinoma, large cell undifferentiated carcinoma, and mixed histologies [2].

Recently, the uptake of 18 F-fluorodeoxyglucose (FDG) as determined by computerized tomography integrated positron emission tomography (PET-CT) has become a widely used non-invasive diagnostic test. Fluorodeoxyglucose PET-CT measures the standardized uptake value (SUV) of a pulmonary mass, which quantifies the glucose avidity of the tumor. Fluorodeoxyglucose PET-CT has been shown to be useful for evaluating an indeterminate pulmonary nodule, staging mediastinal lymph nodes, and evaluating local nodal and distant metastases. Fluorodeoxyglucose uptake correlates with the proliferative activity of tumor and is an independent prognostic factor in patients with lung cancer [3-6].

The objective of the present study is to assess whether the maximum SUV (SUVmax) in PET-CT correlates with tumor size, lymph node metastasis, distant metastasis, and tumor histopathological type in patients with NSCLC.

## Methods

### Study population

The records of 151 patients newly diagnosed with NSCLC between November 2007 and November 2010 were evaluated retrospectively. The subjects were examined by Fluorodeoxyglucose PET-CT and subsequently underwent fiberoptic bronchoscopy for sampling of lymph nodes and histological diagnosis of masses. A total of 139 males and 12 females were included in the study, with a mean age 59 ± 8.40 years (range 34–77 years). Pathologically, there were 41 adenocarcinomas, 45 squamous cell carcinomas, and 65 unspecified NSCLC.

Exclusion criteria were as follows: primary lesion <1 cm, histology confirmed as other than NSCLC, type I diabetes, prior history of lung cancer or other cancer within the previous 5 years, previous therapy or surgical staging for NSCLC before PET, massive or widespread metastatic tumors such that the primary focus could not be identified, and FDG-PET-CT scan performed more than one month prior to tissue diagnosis.

### FGD-PET-CT imaging

All patients underwent diagnostic and/or staging FDG-PET-CT prior to biopsy or therapy. Patients were asked to fast at least 6 h before the FDG-PET-CT scan. All patients had a glucose level below 180 mg/dl and were injected intravenously with 0.22 mCi (8.14 MBq)/kg (10–15 mCi/370–555 MBq) FDG. At 60–90 min after the injection, data were acquired from the vertex to the upper thigh. The first CT scan was performed using 120 kV, 50 mA, and a 3-mm section thickness. Immediately after CT, a PET scan (Siemens Biograph; Siemens Medical Solutions, Inc., Malvern, PA, USA) was performed for about 25 min, with seven to eight bed positions and 3 min/position. PET images were reconstructed iteratively with CT data for attenuation correction, using an inline integrated Siemens Esoft Workstation system. Computerized tomography integrated positron emission tomography fusion images in transaxial, sagittal, and coronal planes were evaluated visually, and the SUVmax of lesions was obtained from transaxial images.

### Statistical evaluation

Statistical analysis was performed using SPSS software (version 12.0). Values were expressed as means ± standard deviation. Statistical significance was assessed at the p < 0.05 level. One-way analysis of variance was performed to compare SUVmax among the histological types. Pearson’s correlations were computed between tumor SUVmax and tumor diameter, lymph node diameter, and lymph node SUVmax. Independent samples *t*-test was used to determine the significance of the difference in tumor SUVmax according to the presence of lymph node or distant metastases.

## Results

The characteristics and SUVmax of the 151 NSCLC cases are summarized in Table 1. A significant relationship was found between tumor SUVmax and tumor size (r = 0.320; p < 0.001). When the cases were divided into three groups based on tumor size (group 1, ≤ 3 cm; group 2, >3 cm and ≤ 5 cm; and group 3, >5 cm), tumor SUVmax did not differ significantly between groups 1 and 2 (p > 0.05), while it was significantly lower in groups 1 and 2 compared with group 3 (p = 0.006 for each). Considering all cases, tumor SUVmax was not significantly correlated with age, gender, or histological type (adenocarcinoma, squamous cell carcinoma, and unspecified NSCLC).

**Table 1:** Characteristics and SUVmax of the NSCLC cases

	**n (%)**	**SUVmax (mean ± SD)**
Sex		
Male	139 (92)	15.33 ± 6.87
Female	12 (8)	11.61 ± 3.66
Histology		
Adenocarcinoma	41 (27)	15.14 ± 7.27
Squamous cell carcinoma	45 (30)	14.40 ± 5.39
Unspecified NSCLC	65 (43)	15.35 ± 7.27
Tumor size		
≤ 3 cm	37 (25)	13.40 ± 6.57
> 3 ≤ 5 cm	69 (45)	13.98 ± 5.90
> 5 cm	45 (30)	17.91 ± 7.27
Lymph node metastases		
N0	127 (84)	14.76 ± 6.72
N2	24 (16)	16.33 ± 9.02
Distant metastasis		
M0	110 (73)	15.08 ± 6.80
M1	41 (27)	14.82 ± 6.62

Among the 151 cases, lymph node metastases were pathologically proven in 24 cases (16%), all of which were N stage 2 (ipsilateral mediastinal lymph node metastasis with or without hilar or intrapulmonary lymph node metastasis). Lymph node metastases were present in 24% (10/41) of adenocarcinomas, 6% (3/45) of squamous cell carcinomas, and 16% (11/65) of unspecified NSCLC. When cases were divided into two groups according to lymph node involvement, there was no difference in tumor SUVmax between the groups. However, lymph node size was positively correlated with lymph node SUVmax (r = 0.775, p < 0.001). Tumor SUVmax did not differ significantly according to the presence of distant metastases.

## Discussion

Although CT or magnetic resonance imaging provides precise anatomical and morphological information, the role of FDG-PET-CT has increased for diagnosis and staging of lung cancer [7]. Recently, FDG uptake has been reported to be a prognostic factor in patients with lung cancer [4-6]. Patz et al. [8] demonstrated that patients with positive FDG-PET-CT results in treated lung cancer had a significantly worse prognosis than patients with negative results. Therefore, we examined whether SUVmax correlates with tumor size, lymph node and distant metastases in patients with NSCLC. Tumor size, but not lymph node or distant metastases, was related to the tumor SUVmax. Doom et al. [9] also reported a strong significant association between tumor size and SUVmax in patients with NSCLC. Another study in patients with stage I NSCLC showed a significant association between the primary tumor, SUVmax and tumor size, with tumors ≤ 3 cm having a significantly lower SUV than tumors > 3 cm [10]. In addition, a retrospective analysis of 85 patients with solid pulmonary lesions found a positive correlation between the size of a malignant tumor and SUVmax [11]. A multivariate analysis demonstrated that the combination of high SUV and large lesion size identified a subgroup of patients with the worst prognosis and a median survival rate of less than 6 months [12].

Aquino et al. [13] reported a significant difference in FDG uptake between the well-differentiated adenocarcinoma subtype bronchioloalveolar carcinoma (BAC) and non-BAC adenocarcinomas, including well-differentiated non-BAC tumors. Adenocarcinomas with mixed features that included BAC had a peak SUV (1.5 ± 0.2) lower than that of all other non-BAC adenocarcinomas (SUV, 3 ± 1.5), which included one poor tumor, three moderate tumors, and one well-differentiated tumor [13]. Vesselle et al. [2] showed that the uptake by large cell carcinomas was greater than that by adenocarcinomas and was not significantly different from uptake by squamous cell carcinomas. However, we observed no difference in SUVmax among histological types. Our data were in concordance with previous studies that documented lower uptake by adenocarcinomas compared with squamous cell carcinomas [10,14-17] and lower uptake by BAC adenocarcinomas compared with non-BAC adenocarcinomas [18-20].

Fluorodeoxyglucose-PET-CT is already an indispensable modality for evaluating lymph node and distant metastases. Many reports have suggested that FDG-PET-CT is superior to CT in the accuracy of N-staging for lung cancer [21-26]. Therefore, FDG-PET-CT is now regarded as the most accurate imaging modality for N-staging of lung cancer. However, a significant number of false-negative and false-positive findings of lung cancer, including N-staging, on FDG-PET-CT have been reported [27-31]. Nambu et al. [32] demonstrated that the likelihood of lymph node metastasis increased with an increase in SUVmax of the primary tumor; for primary lung cancer with a SUVmax greater than 12, the probability of lymph node metastasis was high, reaching 70%, irrespective of the degree of FDG accumulation in the lymph node stations. They concluded that this finding would allow a more sensitive prediction of the presence of lymph node metastases, including the microscopic ones that cannot be detected by direct evaluation of lymph node stations. Consistent with these results, Higashi et al. [33] documented in a multicenter study that the incidence of lymphatic vessel invasion and lymph node metastasis in NSCLC were associated with 18 F-FDG uptake, concluding that 18 F-FDG uptake by a primary tumor is a strong predictor of lymphatic vessel invasion and lymph node metastasis. In the present study, although tumor SUVmax was higher in patients with lymph node metastasis than in those without, the difference did not reach statistical significance. We also observed that the frequency of lymph node metastasis was higher in adenocarcinomas (24%) than in squamous cell carcinomas (6%), suggesting that pathological subtype may be a significant factor associated with lymph node metastasis. In contrast, a previous study showed no difference in the frequency of lymph node metastasis between the two pathological subtypes [32].

Based on univariate analysis, Jeong et al. [14] concluded that metastasis detected by PET imaging, which can affect staging by aiding in the discovery of metastasis to contralateral lymph nodes or distant organs, was an insignificant factor, and that metastatic findings on PET had weak discriminative power. According to Cerfolio et al. [16], FDG-PET-CT does not replace the need for tissue biopsies for staging N1 or N2 lymph nodes, or metastatic lesions, as false positives and false negatives were observed in all stations in their study. However, FDG-PET-CT resulted in better patient selection before pulmonary resection. FDG-PET can also help in targeting areas for biopsy and identifying unsuspected N2 and M1 disease [3]. In the present study, tumor SUVmax was not significantly correlated with distant metastases. This may be attributable to the finding of increased 18 F-FDG uptake by subclinical inflammatory lesions as well as by malignant tumors.

Our study has limitations. As it is a retrospective study, we can report only associations. In addition, we elected to exclude diabetics and patients who received chemotherapy, which might have caused a selection bias.

## Conclusions

SUVmax was associated with tumor size, but not with distant metastases or lymph node involvement. Thus, SUVmax determined by FDG-PET-CT is not predictive of the presence of metastases. Moreover, SUVmax was not related to histological tumor. Larger prospective and randomized analyses may potentially reveal more significant relationships.

## Competing interests

The authors declare that they have no competing interests.
